# Estimation of radiation doses and lifetime attributable risk of radiation-induced cancer in the uterus and prostate from abdomen pelvis CT examinations

**DOI:** 10.3389/fpubh.2022.1094328

**Published:** 2023-01-09

**Authors:** Nasser Shubayr, Yazeed Alashban

**Affiliations:** ^1^Department of Diagnostic Radiography Technology, College of Applied Medical Sciences, Jazan University, Jazan, Saudi Arabia; ^2^Radiological Sciences Department, College of Applied Medical Sciences, King Saud University, Riyadh, Saudi Arabia

**Keywords:** ionizing radiation, cancer incidence, cancer mortality, lifetime attributable risk, computed tomography, uterus, prostate

## Abstract

Computed tomography (CT) scans are one of the most common radiation imaging modalities, and CT scans are rising steadily worldwide. CT has the potential to enhance radiography practice, but it also has the risk of drastically increasing patient doses. One CT procedure for the abdomen pelvis (AP) area can expose a patient's prostate or uterus to a substantial radiation dose, leading to concerns about radiation-induced cancer. This study aimed to estimate organ doses of the uterus and prostate and evaluate the lifetime attributable risk (LAR) of cancer incidence and mortality resulting from AP CT examinations. This retrospective study included 665 patients, of which 380 (57%) were female, and 285 (43%) were male. Data were collected from the picture archiving and communication system for AP CT procedures and exposure parameter data. Organ doses for the uterus and prostate were calculated using National Cancer Institute CT (NCICT) software. Based on the risk models proposed by the BEIR VII report, the calculated organ doses were used to estimate the LAR of prostate and uterus cancer incidence and mortality due to radiation exposure from AP CT procedures. The mean effective dose resulting from AP CT for females and males was 5.76 ± 3.22 (range: 1.13–12.71 mSv) and 4.37 ± 1.66 mSv (range: 1.36–8.07 mSv), respectively. The mean organ dose to the uterus was 10.86 ± 6.09 mGy (range: 2.13–24.06 mGy). The mean organ dose to the prostate was 7.00 ± 2.66 mGy (range: 2.18–12.94 mGy). The LAR of uterus and prostate cancer incidence was 1.75 ± 1.19 cases and 2.24 ± 1.06 cases per 100,000 persons, respectively. The LAR of cancer mortality rates from uterus and prostate cancers were 0.36 ± 0.22 and 0.48 ± 0.18 cases per 100,000 persons, respectively. The LAR of prostate and uterus cancer occurrence and mortality from radiation doses with AP CT procedures was low but not trivial. Therefore, efforts should be made to lower patient doses while retaining image quality. Although the minimization of the patient's radiation dose must guide clinical practice, the estimated slight increase in risk could aid in easing fears regarding well-justified AP CT procedures.

## 1. Introduction

Computed tomography (CT) is a cross-sectional radiation imaging modality that significantly impacts medical diagnosis (1). This modality uses multiple energies and intensities to generate detailed two-dimensional and three-dimensional images and volumetric images of different body parts (2, 3). In recent years, the use of CT has increased considerably, with roughly 70 million CT procedures conducted annually in the USA (4, 5). In other countries, such as the Netherlands, the number of CT procedures doubled from around 580 thousand in 2002 to about 1.16 million in 2010 (1). Organ doses from conventional X-ray procedures are considerably smaller than those associated with CT procedures (6). Accordingly, CT procedures are the main contributors to the collective dose from all medical radiation procedures (7, 8). Although CT procedures make up only 5% of all X-ray exams, they contribute 40–67% of the overall medical dose [9] and are predicted to increase by around 10–15% yearly (9). Epidemiological studies have linked low levels of exposure to ionizing radiation in medical imaging procedures to the development of cancer and radiation-related diseases (10, 11).

Low-dose radiation risk is controversial, with claims that low-dose risks are overestimated (12) or underestimated (13, 14) using linear extrapolation from moderate-dose exposed groups. The linear no-threshold (LNT) model of ionizing radiation–induced cancer assumes that every increment of radiation dose, no matter how small, constitutes an increased cancer risk for humans. The assumption underlying the LNT model, frequently adopted by expert advisory bodies (15), that the risk at low doses is nearly linear with dose is a question of whether a low-dose risk exists. Although there is some radiobiological support for LNT based on DNA damage considerations, it is acknowledged to be an estimate made for practicality in the context of radiological protection. In fact, a substantial body of evidence suggests that there is considerable evidence of cancer risk at low doses (16, 17).

The survivors of the atomic bombings in Hiroshima and Nagasaki are one of the most important sources of information on radiation risks. Long-term survivors of the Hiroshima and Nagasaki atomic bombs, who were exposed to radiation doses, have been shown to have an increased risk of cancer (18–21). In a follow-up from 1958 to 2009, recent studies investigated the incidence of prostate and uterine cancer in a cohort of atomic bomb survivors from the Life Span Study, which included 62,534 women and 41,544 men. A study demonstrated a substantial linear dose response for prostate cancer, with an estimated excess relative risk (ERR) per Gy of 0.57 (22). The study concluded that “the observed dose response strengthens the evidence of a radiation effect on the risk of prostate cancer incidence in the atomic bomb survivors” (22). In addition, an increased risk of prostate cancer has been found after X-ray treatment for ankylosing spondylitis (23) and in a subset of nuclear workers who were internally exposed to various radionuclides (24). The study of female atomic bomb survivors found a significant association between radiation dose and risk of uterine cancer (ERR/Gy of 0.73), especially for exposure occurring in mid-puberty, but not for either early childhood or adult exposures (25). Increased corpus cancer risk has been reported in several studies of high-dose radiotherapy patients (26–28) and in one study of radiation workers (29).

In abdominal pelvic (AP) CT, the prostate and uterus are exposed to direct radiation, which can pose health risks to patients. Even though the radiation risk to any given patient may be low, the growing number of persons exposed and the rising radiation dose per procedure could lead to a significant number of cancer incidents directly related to radiation exposure from CT. It is crucial to understand how much radiation is supplied to patients during CT procedures to properly balance the probability of harm and the potential benefits. This is especially important because the threshold for using CT has been lowered and is now being used more frequently on healthy individuals in whom the risk of potential CT-induced carcinogenesis may outweigh its diagnostic usefulness. Therefore, this study aimed to estimate organ doses of the uterus and prostate and evaluate the lifetime attributable risk (LAR) of cancer incidence and mortality resulting from AP CT examinations.

## 2. Materials and methods

### 2.1. Study design

This retrospective study was conducted over 1 year at two general hospitals in Jazan, Saudi Arabia. The radiology departments included in this study were equipped with Siemens Somatom 64 CT scanners and GE Lightspeed 16 CT scanners. This study was approved by the ethics committee of Jazan University in Jazan, Saudi Arabia (approval number: REC/44/788).

### 2.2. Study population and data collection

The study population was comprised of adult patients. Patient demographic data, including age and gender, were collected. Data were collected from the picture archiving and communication system (PACS). The collected data included the AP CT and exposure parameter data [CT dose index (CTDIvol) and dose-length product (DLP), and scan length]. Incomplete examinations, such as those missing one or more acquisitions, were excluded from the analysis.

### 2.3. CT dosimetry

The National Cancer Institute CT (NCICT) dosimetry tool was used to calculate the organ doses of the prostate and uterus. The NCICT dosimetry tool is a massive library of precomputed dose factors for various computational phantoms linked with Monte Carlo radiation transport methodologies (30). The dose factors (milligray/milligray) were calculated while considering various phantom sizes, which are the organ-absorbed dose (millgrays) normalized to the CTDIvol (millgrays) of the reference scanner. The absolute organ doses were estimated by multiplying the dose factors by the reported CTDIvol (milligrays) for each CT scanner of interest (milligrays). Lee et al. (30) reported on the intricate methods employed in organ dose calculations. The effective dose resulting from AP CT scans was also obtained using the NCICT.

### 2.4. Radiation risk assessment

By extrapolating from the risk estimated at high doses, LNT model has been the standard risk assessment utilized by the radiation protection community to determine the health effects associated with low doses (15, 31, 32). The risk models proposed by the BEIR VII (2006) report describe a technique to approximate the LAR of cancer based on the amount of a single radiation dose and a patient's age (32). LAR is defined as an additional cancer risk above and beyond baseline cancer risk. The age- and sex-specific LAR of the uterus and prostate cancer incidence and mortality for organ doses were calculated using BEIR VII risk estimates.

### 2.5. Statistical analysis

Statistical analysis was performed using the Statistical Package for the Social Sciences (SPSS version 20, IBM, Somers, NY, USA). Descriptive statistics of the continuous data were presented as mean ± SD. Inferential statistical tests, independent samples *t*-tests and one-way analysis of variance (ANOVA), were performed to determine if gender and age groups are significantly different from each other on CTDIvol, DLP, scan length, effective and organ doses, with *p* < 0.05 considered statistically significant.

## 3. Results

### 3.1. Demographic and CT acquisition parameters

As illustrated in Table 1, the study included 665 CT procedures for adult patients with 380 (57%) females [mean age, 43.58 ± 17.74 years; range, 18–82 years], and 285 (43%) males [mean age, 42.79 ± 15.69 years; range, 18–80 years]). The AP CT were distributed among age groups as 31% for 18–35 years, 39% for 36–55 years, and 30% for >55 years. The mean DLP and CTDIvol values were higher for females compared to male patients, with no statistically significant differences. The mean scan length for male patients (49.75 ± 14.89 cm) was higher than for females (41.77 ± 11.92 cm), with statistically significant differences (*p* = 0.001).

**Table 1:** Mean values ± SD of CTDIvol, DLP, and scan length for AP CT examinations.

**Gender**	**Age groups**	**N (%)**	**CTDIvol (mGy)**	**DLP (mGy.cm)**	**Scan length (cm)**
Female	18–35	125	13.37 ± 7.2	611.32 ± 368.38	44.64 ± 13.17
	36–55	140	13.61 ± 8.12	537.07 ± 359.49	39.08 ± 11.73
	>55	115	17.67 ± 8.93	799.3 ± 497.16	41.92 ± 10.38
	Overall	380 (57)	14.76 ± 8.21	640.86 ± 417.69	41.77 ± 11.92
Male	17–35	80	10.93 ± 5.57	531.13 ± 310.31	47.52 ± 14.93
	36–55	120	13.72 ± 4.51	669 ± 246.84	50.04 ± 12.96
	>55	85	12.17 ± 3.28	657.64 ± 336.27	52.35 ± 19.28
	Overall	285 (43)	12.51 ± 4.73	623.29 ± 288.98	49.75 ± 14.89

### 3.2. Effective and organ dose estimations

**Figure 1:**
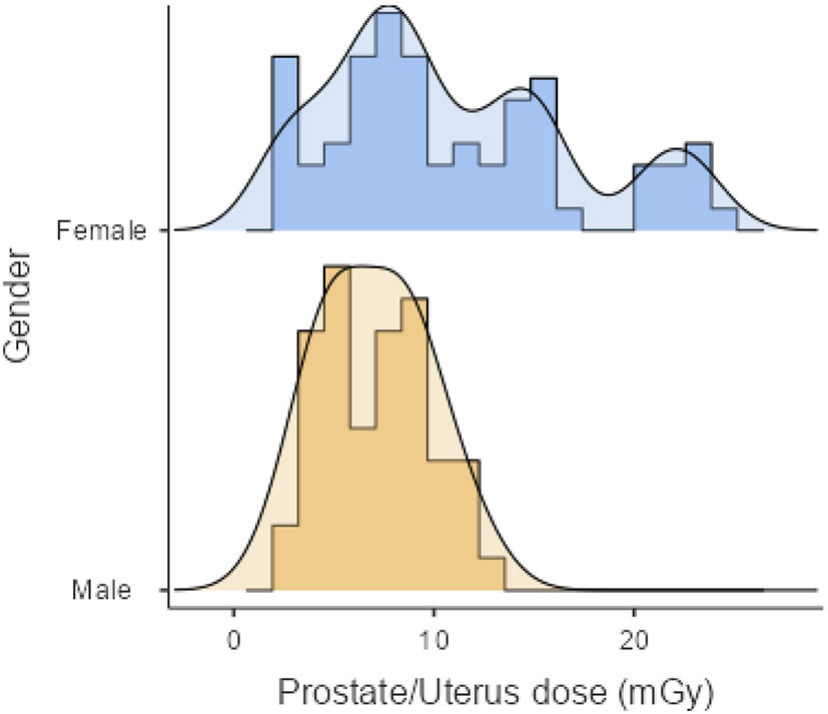
Distribution of organ doses to the uterus and prostate from AP CT examinations.

As shown in Table 2, the mean effective doses resulting from AP CT for females and males were 5.76 ± 3.22 (range: 1.13–12.71 mSv) and 4.37 ± 1.66 mSv (range: 1.36–8.07 mSv), respectively. Mean organ doses for older age groups were higher than younger age groups, with statistically significant differences (*p* < 0.001). The mean organ dose to the uterus was 10.86 ± 6.09 mGy (range: 2.13–24.06 mGy). The mean organ dose to the prostate was 7.00 ± 2.66 mGy (range: 2.18–12.94 mGy). The effective and organ doses were higher for female patients than for male patients, with statistically significant differences (*p* = 0.005). The distribution of organ doses for female and male patients is illustrated in Figure 1.

**Table 2:** Mean values ± SD of effective dose and organ dose to the uterus and prostate from AP CT examinations.

**Age groups**	**17–35**	**36–55**	**>55**	**Overall**
**Female**				
Uterus dose (mGy)	9.79 ± 5.21	9.99 ± 6.05	13.07 ± 6.68	10.86 ± 6.09
				(2.13–24.06)
Effective dose (mSv)	5.19 ± 2.75	5.31 ± 3.21	6.93 ± 3.53	5.76 ± 3.22
				(1.13–12.71)
**Male**				
Prostate dose (mGy)	6.11 ± 3.11	7.65 ± 2.55	6.83 ± 1.89	7.00 ± 2.66
				(2.18–12.94)
Effective dose (mSv)	3.82 ± 1.94	4.78 ± 1.59	4.26 ± 1.18	4.37 ± 1.66
				(1.36–8.07)

### 3.3. Individual radiation risk assessment

**Figure 2:**
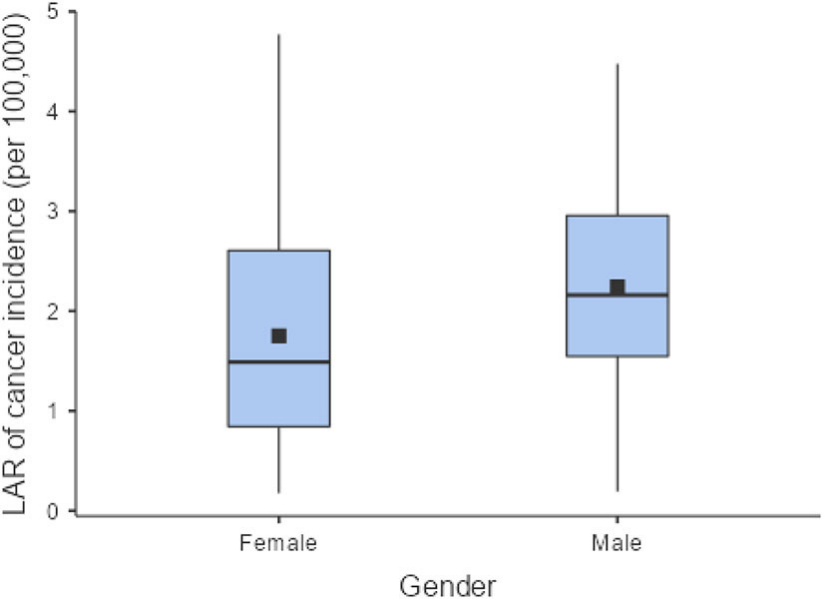
LAR of prostate and uterus cancer incidence for female and male patients.

**Figure 3:**
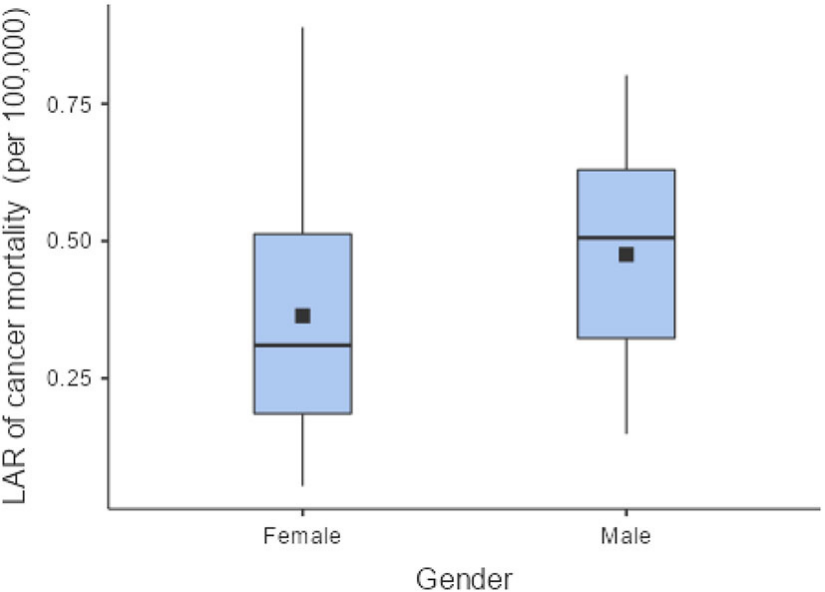
The LAR of prostate and uterus cancer mortality for female and male patients.

**Figure 4:**
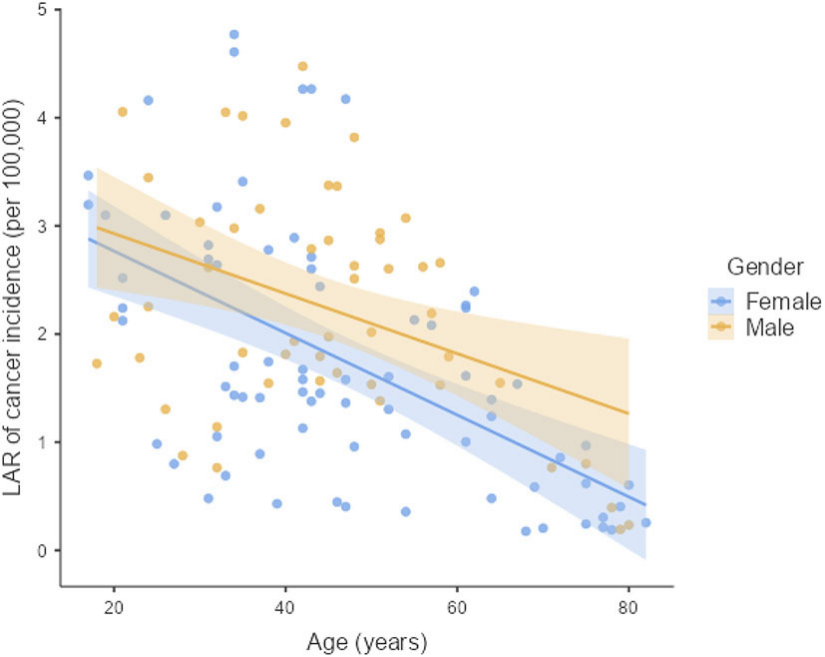
LAR values of prostate and uterus cancer incidence from AP CT as a function of age and gender.

**Figure 5:**
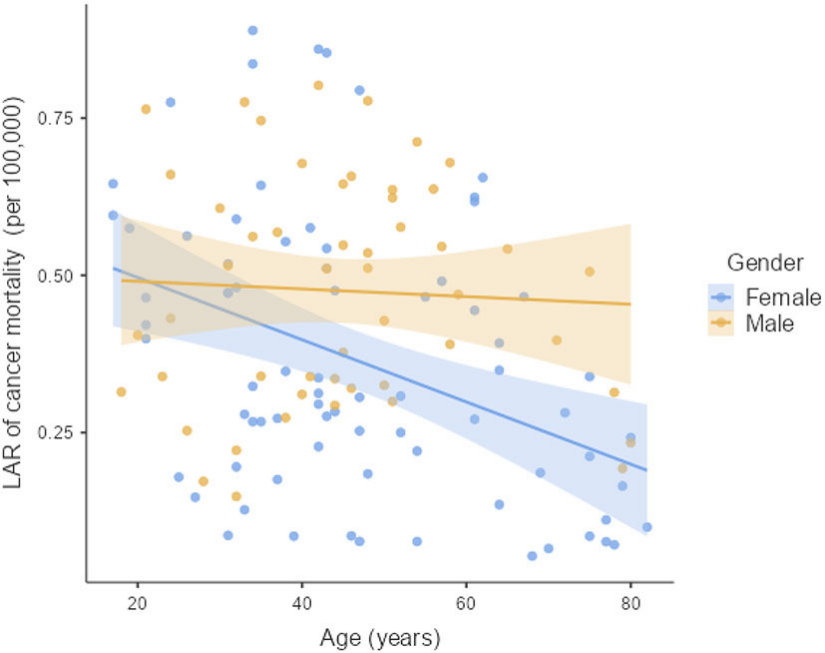
LAR values of prostate and uterus cancer mortality from AP CT as a function of age and gender.

The LAR of uterus and prostate cancer incidence was 1.75 ± 1.19 cases and 2.24 ± 1.06 cases per 100,000 persons, respectively (Figure 2). The LAR of cancer mortality from uterus and prostate cancers were 0.36 ± 0.22 and 0.48 ± 0.18 cases per 100,000 persons, respectively (Figure 3). The values of LAR of prostate and uterus cancer occurrence from AP CT as a function of age and gender were higher in male patients than in females, with a consistent decline with age at exposure (Figure 4). When examining the values of LAR of prostate and uterine cancer mortality as a function of age and gender, it was shown that male and female patients both had a minor decline in LAR with increasing age at exposure (Figure 5).

## 4. Discussion

CT scans are one of the most common radiation imaging modalities, and CT scans are rising steadily worldwide. CT scans are constantly increasing worldwide with the potential to improve the practice of radiography, but they also have the potential to increase the patient dose. A single CT scan of the abdomen pelvis (AP) can expose a patient's prostate or uterus to a substantial radiation dose, leading to rising concerns about radiation-induced cancer. However, medical staff may not have adequate knowledge of the risks of ionizing radiation used in these procedures. The risk of cancer incidence and mortality from ionizing radiation is appropriately expressed in terms of LAR values. Generally, radiation doses as low as reasonably achievable and consistent with acceptable image quality remain the most significant strategies for diminishing this potential risk. This study evaluated organ doses of the uterus and prostate and the LAR of cancer incidence and mortality resulting from AP CT.

The mean scan length for male patients was higher than for females, with statistically significant differences (*p* < 0.001). These results are consistent with the statistical studies showing that Saudi males' body height is relatively higher than that of Saudi females (33, 34). The results show that the mean value of effective dose (female: 5.76 ± 3.22 mSv vs. male: 4.37 ± 1.66 mSv) and organ dose (female: 10.86 ± 6.09 mGy vs. male: 7.00 ± 2.66 mGy) and DLP (female: 640.86 ± 417.69 mGy.cm vs. male: 623.29 ± 288.98 mGy.cm) were higher for females than for males. This is primarily because the DLP values were higher for females than for male patients. Patrick et al. investigated multiple body composition parameters to determine the strongest predictor of effective doses among patients. Their study found that total adipose tissue volume was significantly higher in females compared to male patients, and adipose tissue volume was the strongest predictor of DLP (35). Studies among the Saudi population have reported that Saudi females have higher body weight and cross-sectional area than males (36, 37). In comparison with other studies, the effective dose and organ doses to the prostate and uterus from AP CT in our study were lower than previously reported in other studies: 13 mSv (38) and 13.6 (39) for effective doses, and organ doses to the uterus of 10.4 mGy and the prostate of 10 mGy (40).

The LAR values of cancer incidence and mortality from AP CT were obtained for the prostate (2.24 and 0.48 cases per 100,000 persons) and the uterus (1.75 ± 1.19 cases and 0.36 ± 0.22 cases per 100,000 persons), indicating that males are at higher risk than females. The LAR of uterus cancer incidence and mortality as a function of age showed a steadily declining risk with age at exposure (Figures 4, 5). The LAR of prostate cancer incidence decreased with age at exposure. However, unlike for females in this study, the LAR of cancer mortality for prostate cancer showed a slightly decreasing trend with age at exposure for male patients (Figure 5). This is because the LAR of cancer mortality for the prostate remains within 7 (±2) cases from age 30 to 80 years, and the organ doses of the prostate dose were higher for older age groups in this study. Prostate cancer is among the most common cancers in males, with high morbidity and mortality in Saudi Arabia, with more prevalence in the age group of 50–70 years (41). The increased prostate organ doses and the natural prevalence of prostate cancer for older groups require special attention for male patients in Saudi Arabia.

Radiation has a stochastic effect that can cause cancer; no threshold value or dose can cause it (42). Calculating risk does not mean identifying a risk unique to each patient (43). Nonetheless, the LAR estimate offers a standard for patients, radiation oncologists, and other medical personnel. As a result, this study's findings may help develop a database for calculating LARs related to CT scans of the pelvis.

Specific dose reduction strategies for reducing patient radiation dose from CT examination, such as tube current (mA) modulation, iterative reconstruction techniques, staff awareness, and the use of advanced imaging technologies, are found in the literature. Consequently, radiation protection during CT examination is essential, regardless of the radiation dose received (44–47).

This study has some limitations. First, there are differences between the Saudi population's baseline cancer incidence levels and mortality rates and those listed in the BEIR VII study (32). Second, the LAR of cancer occurrence from X-ray ranged from 80–140 kVp, possibly distinct from that of high-energy gamma rays that the BEIR VII database represents a majority based on these high-energy rays (48).

## 5. Conclusions

Using the risk models proposed by the BEIR VII report, the LAR of prostate and uterus cancer incidence and mortality due to radiation exposure from AP CT procedures were low, but not trivial. Moreover, risk associated with prostate cancer from radiation exposure along with the high natural prevalence of prostate cancer among older people necessitate special attention. Therefore, efforts should be made to reduce patient doses while maintaining image quality. Although the minimization of the patient's radiation dose must guide clinical practice, the estimated small increase in risk could aid in easing fears regarding well-justified AP CT procedures. To reduce the patients' LARs, different treatment planning CT protocols should be optimized to reduce the radiation dose.

## Data availability statement

The raw data supporting the conclusions of this article will be made available by the authors, without undue reservation.

## Ethics statement

The studies involving human participants were reviewed and approved by Jazan University. Written informed consent for participation was not required for this study in accordance with the national legislation and the institutional requirements.

## Author contributions

All authors listed have made a substantial, direct, and intellectual contribution to the work and approved it for publication.

## References

[1] van der Molen AJ Schilham A Stoop P Prokop M Geleijns J A. national survey on radiation dose in CT in the Netherlands. Insights Imag. (2013) 4:383–90. 10.1007/s13244-013-0253-9 23673455 PMC3675255

[2] Copley DC Eberhard JW Mohr GA. Computed tomography part I: Introduction and industrial applications. JOM. (1994) 46:14–26.

[3] Suetens P. Fundamentals of Medical Imaging. Cambridge: Cambridge University Press (2017). 10.1017/9781316671849

[4] Ogbole GI. Radiation dose in paediatric computed tomography: risks and benefits. Ann Ib Postgrad Med. (2010) 8:118–26. 10.4314/aipm.v8i2.71823 25161479 PMC4111023

[5] Smith-Bindman R Lipson J Marcus R Kim KP Mahesh M Gould R. Radiation dose associated with common computed tomography examinations and the associated lifetime attributable risk of cancer. Arch Intern Med. (2009) 169:2078–86. 10.1001/archinternmed.2009.427 20008690 PMC4635397

[6] Brenner DJ Hall EJ. Computed tomography: an increasing source of radiation exposure. N Engl J Med. (2007) 357:2277–84. 10.1056/NEJMra072149 18046031

[7] Nickoloff EL Alderson PO. A comparative study of thoracic radiation doses from 64-slice cardiac CT. Br J Radiol. (2007) 80:537–44. 10.1259/bjr/34603706 17704315

[8] Johnson JN Hornik CP Li JS Benjamin DK Yoshizumi TT Reiman RE. Cumulative radiation exposure and cancer risk estimation in children with heart disease. Circulation. (2014) 130:161–7. 10.1161/CIRCULATIONAHA.113.005425 24914037 PMC4103421

[9] Linton OW Mettler FA National Council on Radiation P Measurements. National conference on dose reduction in CT, with an emphasis on pediatric patients. AJR Am J Roentgenol. (2003) 181:321–9. 10.2214/ajr.181.2.1810321 12876005

[10] Rehani MM Yang K Melick ER Heil J Salat D Sensakovic WF. Patients undergoing recurrent CT scans: assessing the magnitude. Eur Radiol. (2020) 30:1828–36. 10.1007/s00330-019-06523-y 31792585

[11] Sodickson A Baeyens PF Andriole KP Prevedello LM Nawfel RD Hanson R. Recurrent CT, cumulative radiation exposure, and associated radiation-induced cancer risks from CT of adults. Radiology. (2009) 251:175–84. 10.1148/radiol.2511081296 19332852

[12] Cuttler J. Can we abolish the 60-year-old international consensus that connects nuclear radiation to cancer? Nucl News. (2022) 65:46.

[13] Schmitz-Feuerhake I Pflugbeil S. 'Lifestyle' and cancer rates in former East and West Germany: the possible contribution of diagnostic radiation exposures. Radiat Protect Dosimetry. (2011) 147:310–3. 10.1093/rpd/ncr348 21835840

[14] Kneale G Stewart A Mancuso T. Radiation exposures of hanford workers dying from cancer and other causes. Health Phys. (1979) 36:87. 422389

[15] ICRP. The 2007 Recommendations of the International Commission on Radiological Protection. Ottawa: ICRP publication (2007).

[16] Simon S Kendall G Bouffler S Little M. The evidence for excess risk of cancer and non-cancer disease at low doses and dose rates. Radiat Res. (2022) 198:615–24. 10.1667/RADE-22-00132.1 36136740 PMC9797580

[17] Berrington de Gonzalez A Pasqual E Veiga L. Epidemiological studies of CT scans and cancer risk: the state of the science. Br J Radiol. (2021) 94:20210471. 10.1259/bjr.20210471 34545766 PMC9328069

[18] Brenner DJ Doll R Goodhead DT Hall EJ Land CE Little JB. Cancer risks attributable to low doses of ionizing radiation: assessing what we really know. Proc Natl Acad Sci. (2003) 100:13761–6. 10.1073/pnas.2235592100 14610281 PMC283495

[19] Preston DL Pierce DA Shimizu Y Ron E Mabuchi K. Dose response and temporal patterns of radiation-associated solid cancer risks. Health Phys. (2003) 85:43–6. 10.1097/00004032-200307000-00010 12852470

[20] Preston DL Ron E Tokuoka S Funamoto S Nishi N Soda M. Solid cancer incidence in atomic bomb survivors: 1958–1998. Radiat Res. (2007) 168:1–64. 10.1667/RR0763.1 17722996

[21] Pierce DA Preston DL. Radiation-related cancer risks at low doses among atomic bomb survivors. Radiat Res. (2000) 154:178–86. 10.1667/0033-7587(2000)154[0178:RRCRAL]2.0.CO;2 10931690

[22] Mabuchi K Preston DL Brenner AV Sugiyama H Utada M Sakata R. Risk of prostate cancer incidence among atomic bomb survivors: 1958–2009. Radiat Res. (2021) 195:66–76. 10.1667/RR15481.1 33181833 PMC7849930

[23] Weiss HA Darby SC Doll R. Cancer mortality following X-ray treatment for ankylosing spondylitis. Int J Cancer. (1994) 59:327–38. 7927937 10.1002/ijc.2910590307

[24] Rooney C Beral V Maconochie N Fraser P Davies G. Case-control study of prostatic cancer in employees of the United Kingdom atomic energy authority. BMJ. (1993) 307:1391–7. 8274891 10.1136/bmj.307.6916.1391 PMC1679658

[25] Utada M Brenner AV Preston DL Cologne JB Sakata R Sugiyama H. Radiation risks of uterine cancer in atomic bomb survivors: 1958–2009. JNCI Cancer Spect. (2018) 2:pky081. 10.1093/jncics/pky081 31249993 PMC6586771

[26] Boice JD Engholm G Kleinerman RA Blettner M Stovall M Lisco H. Radiation dose and second cancer risk in patients treated for cancer of the cervix. Radiat Res. (1988) 116:3–55. 3186929

[27] Sakata R Kleinerman RA Mabuchi K Stovall M Smith SA Weathers R. Cancer mortality following radiotherapy for benign gynecologic disorders. Radiat Res. (2012) 178:266–79. 10.1667/RR2845.1 22856888 PMC3471655

[28] Inskip PD Monson RR Wagoner JK Stovall M Davis FG Kleinerman RA. Cancer mortality following radium treatment for uterine bleeding. Radiat Res. (1990) 123:331–44. 2217730

[29] Atkinson WD Law DV Bromley KJ Inskip HM. Mortality of employees of the United Kingdom atomic energy authority, 1946–1997. Occup Environ Med. (2004) 61:577–85. 10.1136/oem.2003.012443 15208373 PMC1740809

[30] LeeCKimKPBolchWEMorozBEFolioL. NCICT a computational solution to estimate organ doses for pediatric and adult patients undergoing CT scans. J Radiol Protect Off J Soc Radiol Protect. (2015) 35:891–909. 10.1088/0952-4746/35/4/89126609995

[31] Brenner DJ Sachs RK. Estimating radiation-induced cancer risks at very low doses: rationale for using a linear no-threshold approach. Radiat Environ Biophys. (2006) 44:253–6. 10.1007/s00411-006-0029-4 16470411

[32] Council NR. Health Risks from Exposure to Low Levels of Ionizing Radiation: BEIR VII Phase 2. Virginia: Council NR (2006), p. 1–406.

[33] Khalid ME Ali ME. Relationship of body weight to altitude in Saudi Arabia. Ann Saudi Med. (1994) 14:300–3. 17586923 10.5144/0256-4947.1994.300 PMC6363518

[34] Rodriguez-Martinez A Zhou B Sophiea MK Bentham J Paciorek CJ Iurilli ML. Height and body-mass index trajectories of school-aged children and adolescents from 1985 to 2019 in 200 countries and territories: a pooled analysis of 2181 population-based studies with 65 million participants. Lancet. (2020) 396:1511–24. 10.1016/S0140-6736(20)31859-6 33160572 PMC7658740

[35] McLaughlin PD Chawke L Twomey M Murphy KP O'Neill SB McWilliams SR. Body composition determinants of radiation dose during abdominopelvic CT. Insights Imag. (2018) 9:9–16. 10.1007/s13244-017-0577-y 29063481 PMC5825306

[36] Al-Quwaidhi AJ Pearce MS Critchley JA Sobngwi E O'Flaherty M. Trends and future projections of the prevalence of adult obesity in Saudi Arabia, 1992–2022. East Mediterr Health J. (2014) 20:589–95. 10.26719/2014.20.10.589 25356689

[37] Moradi-Lakeh M El Bcheraoui C Tuffaha M Daoud F Al Saeedi M Basulaiman M. The health of Saudi youths: current challenges and future opportunities. BMC Fam Pract. (2016) 17:26. 10.1186/s12875-016-0425-z 26946327 PMC4779578

[38] Shrimpton PC Jansen JT Harrison JD. Updated estimates of typical effective doses for common CT examinations in the UK following the 2011 national review. Br J Radiol. (2016) 89:20150346. 10.1259/bjr.20150346 26544160 PMC4985946

[39] Osei EK Darko J A. survey of organ equivalent and effective doses from diagnostic radiology procedures. ISRN Radiol. (2013) 2013:204346. 10.5402/2013/204346 24977137 PMC4045519

[40] Almasri H Inayyem W. Evaluation of radiation doses for patients undergoing abdominopelvic computed tomography examination in palestine. Jpn J Health Phys. (2021) 56:75–9. 10.5453/jhps.56.75

[41] Althubiti MA Nour Eldein MM. Trends in the incidence and mortality of cancer in Saudi Arabia. Saudi Med J. (2018) 39:1259–62. 10.15537/smj.2018.12.23348 30520511 PMC6344657

[42] Majewska N Stanisic MG Blaszak MA Juszkat R Frankiewicz M Krasinski Z. Clinical factors increasing radiation doses to patients undergoing long-lasting procedures: abdominal stent-graft implantation. Med Sci Monit Int Med J Exp Clin Res. (2011) 17:Mt97–103. 10.12659/MSM.882033 22037751 PMC3539495

[43] Aw-Zoretic J Seth D Katzman G Sammet S. Estimation of effective dose and lifetime attributable risk from multiple head CT scans in ventriculoperitoneal shunted children. Eur J Radiol. (2014) 83:1920–4. 10.1016/j.ejrad.2014.07.006 25130177 PMC4623705

[44] McCollough C Bruesewitz M Kofler J CT. dose reduction and dose management tools: overview of available options1. Radiogr Rev Publ Radiol Soc N Am. (2006) 26:503–12. 10.1148/rg.262055138 16549613

[45] Yu L Liu X Leng S Kofler JM Ramirez-Giraldo JC Qu M. Radiation dose reduction in computed tomography: techniques and future perspective. Imag Med. (2009) 1:65–84. 10.2217/iim.09.5 22308169 PMC3271708

[46] Lasio G Whiting B Williamson J. Statistical reconstruction for x-ray computed tomography using energy-integrating detectors. Phys Med Biol. (2007) 52:2247–66. 10.1088/0031-9155/52/8/014 17404467

[47] Deak P Langner O Lell M Kalender W. Effects of adaptive section collimation on patient radiation dose in multisection spiral CT. Radiology. (2009) 252:140–7. 10.1148/radiol.2522081845 19561253

[48] Kim S Yoshizumi T Frush D Toncheva G Yin F-F. Radiation dose from cone beam CT in a pediatric phantom: risk estimation of cancer incidence. AJR Am J Roentgenol. (2010) 194:186–90. 10.2214/AJR.08.2168 20028922

